# *Notes from the Field:* Emergency Department Visits for Nonfatal Pedal Cyclist Injuries Before and During the COVID-19 Pandemic, United States, 2019–2020

**DOI:** 10.15585/mmwr.mm7228a3

**Published:** 2023-07-14

**Authors:** Livia Navon, Keming Yuan, Laurie Beck

**Affiliations:** 1 Division of Injury Prevention, National Center for Injury Prevention and Control, CDC.

During the early months of the COVID-19 pandemic, many jurisdictions implemented stay-at-home orders (*1*). Vehicle miles traveled (VMT)* in April 2020 declined by 40% compared with VMT in April 2019; annual VMT in 2020 declined by 13% compared with those in 2019 (*2*). Despite decreased VMT, pedal cyclist traffic crash fatalities increased by 10% from 859 in 2019 to 948 in 2020 (*3*). In 2021, pedal cyclist fatalities increased to 966, the highest number reported since 1975 (*3*,*4*). Given the increase in pedal cyclist fatalities despite the decline in VMT in 2020, emergency department (ED) visits for nonfatal pedal cyclist injuries in 2019 and 2020 were compared.

## Investigation and Outcomes

ED visits for nonfatal pedal cyclist injuries^†^ were identified from the 2019–2020 National Electronic Injury Surveillance System–All Injury Program (NEISS-AIP). NEISS-AIP data are collected from a stratified probability sample of hospitals and provide weighted national estimates of ED visits for nonfatal injuries. The monthly proportions of injury-related ED visits accounted for by pedal cyclist injuries in 2020 and 2019 were compared using pairwise t-tests in SAS-callable SUDAAN (version 11.0.3; RTI International); comparison of the changes in monthly proportions by age group and sex was assessed using logistic regression. Variance was estimated using Taylor series linearization. This activity was reviewed by CDC and was conducted consistent with applicable federal law and CDC policy.^§^

During the early months of the COVID-19 pandemic (March–April 2020), ED visits for nonfatal injuries declined by 31% compared with March–April 2019; the total number of nonfatal injury–related ED visits in 2020 declined by 15% compared with 2019. Despite the decline in total injury-related ED visits, the number of ED visits for pedal cyclist injuries in 2020 (356,630 visits [95% CI = 265,330–447,931]) was 8% higher than in 2019 (328,903 visits [95% CI = 255,096–402,711]). During March–August 2020 and in November 2020, monthly proportions of injury-related ED visits accounted for by pedal cyclist injuries were significantly higher than during the same months in 2019 (Table). The age group with the largest increase during most months was children and adolescents aged <18 years. For example, pedal cyclist injuries in this age group accounted for 6.0% of injury-related ED visits in April 2020, which was 2.9 times higher than in April 2019 (2.1%). In April 2020, pedal cyclist injuries among adults aged ≥18 years accounted for 1.5% of injury-related ED visits, which was 1.5 times higher than in April 2019 (1.0%); among adults aged ≥50 years, the proportion of pedal cyclist ED visits in April 2020 (1.7%) was 2.1 times higher than in April 2019 (0.8%). Increases among children and adolescents aged <18 years were sustained during February–November 2020; among adults aged ≥18 years, increased monthly proportions of pedal cyclist ED visits were observed primarily during March–June 2020.

**TABLE:** Estimated monthly number of emergency department visits for total and pedal cyclist–related nonfatal injuries and monthly percentage of visits due to pedal cyclist injuries, by age group and sex — National Electronic Injury Surveillance System–All Injury Program, United States, 2019–2020

Characteristic/Yr	Pedal cyclist–related injury ED visits											
	Jan	Feb	Mar	Apr	May	Jun	Jul	Aug	Sep	Oct	Nov	Dec
**Age group, yrs, % (95% CI) **												
**2019**												
<18	0.5 (0.3–0.8)	0.8 (0.3–1.2)	1.3 (0.9–1.8)	2.1 (1.7–2.5)	2.8 (2.3–3.3)	3.7 (3.2–4.2)	3.1 (2.5–3.6)	3.9 (3.0–4.8)	2.8 (2.2–3.3)	1.6 (1.2–1.9)	1.0 (0.7–1.4)	0.8 (0.4–1.3)
≥18	0.7 (0.3–1.1)	0.7 (0.4–1.0)	0.8 (0.4–1.1)	1.0 (0.6–1.3)	1.0 (0.8–1.3)	1.1 (1.0–1.3)	1.4 (1.1–1.6)	1.3 (1.1–1.5)	1.3 (1.0–1.5)	1.1 (0.8–1.4)	0.8 (0.5–1.0)	0.7 (0.4–0.9)
18–49	0.7 (0.3–1.0)	0.5 (0.3–0.7)	0.7 (0.4–0.9)	1.1 (0.7–1.4)	1.0 (0.7–1.2)	1.2 (1.0–1.4)	1.4 (1.1–1.7)	1.4 (1.1–1.6)	1.5 (1.1–1.8)	1.1 (0.8–1.4)	0.7 (0.5–0.9)	0.6 (0.4–0.9)
≥50	—*	0.9 (0.4–1.4)	—	0.8 (0.4–1.3)	1.1 (0.8–1.4)	1.0 (0.8–1.3)	1.3 (1.1–1.6)	1.2 (0.8–1.6)	1.0 (0.7–1.3)	1.0 (0.7–1.4)	0.9 (0.5–1.3)	0.7 (0.3–1.1)
**2020**												
<18	0.7 (0.4–1.0)	1.2^†^ (0.8–1.7)	2.2^†^ (1.7–2.7)	6.0^†^ (4.8–7.2)	5.7^†^ (4.8–6.6)	5.4^†^ (4.4–6.5)	5.3^†^ (4.6–6.1)	5.1^†^ (4.1–6.1)	3.9^†^ (2.9–4.9)	2.4^†^ (1.9–2.8)	1.8^†^ (1.3–2.2)	1.1 (0.7–1.5)
≥18	0.8 (0.4–1.2)	0.8 (0.4–1.1)	1.1^†^ (0.6–1.7)	1.5^†^ (0.9–2.0)	1.7^†^ (1.2–2.2)	1.5^†^ (1.2–1.8)	1.4 (1.1–1.7)	1.5^†^ (1.3–1.7)	1.4 (1.2–1.6)	1.2 (0.9–1.5)	0.9 (0.6–1.2)	0.7 (0.4–1.1)
18–49	0.8 (0.4–1.1)	0.7 (0.4–1.0)	0.9^†^ (0.6–1.3)	1.2 (0.8–1.7)	1.5^†^ (1.1–2.0)	1.4 (1.1–1.7)	1.5 (1.1–1.8)	1.6 (1.2–1.9)	1.4 (1.2–1.6)	1.3 (0.9–1.6)	0.8 (0.5–1.0)	0.7 (0.4–0.9)
≥50	—	0.9 (0.4–1.4)	—	1.7^†^ (0.9–2.6)	1.9 (1.0–2.8)	1.6^†^ (1.2–2.0)	1.4 (1.0–1.7)	1.4 (1.1–1.7)	1.3 (1.0–1.7)	1.2 (0.8–1.5)	1.1 (0.6–1.5)	—
**Sex, % (95% CI)**												
**2019**												
Female	0.4 (0.2–0.6)	—	0.4 (0.2–0.7)	0.7 (0.4–1.0)	0.8 (0.6–1.0)	1.0 (0.8–1.2)	0.9 (0.7–1.1)	0.8 (0.7–1.0)	0.8 (0.6–1.1)	0.6 (0.4–0.9)	0.3 (0.2–0.5)	0.3 (0.2–0.5)
Male	0.9 (0.5–1.4)	0.9 (0.5–1.2)	1.2 (0.8–1.7)	1.6 (1.3–1.9)	2.0 (1.7–2.3)	2.2 (1.9–2.4)	2.3 (2.0–2.6)	2.6 (2.2–2.9)	2.2 (1.8–2.5)	1.6 (1.4–1.9)	1.3 (0.9–1.6)	1.0 (0.6–1.5)
**2020**												
Female	0.5 (0.2–0.8)	0.5 (0.2–0.7)	0.9^†^ (0.5–1.3)	1.7^†^ (1.1–2.3)	1.7^†^ (1.2–2.2)	1.5^†^ (1.3–1.7)	1.4^†^ (1.1–1.6)	1.4^†^ (1.1–1.7)	1.1 (0.8–1.3)	0.7 (0.5–1.0)	0.5 (0.3–0.8)	—
Male	1.0 (0.5–1.5)	1.2^†^ (0.7–1.7)	1.6^†^ (1.1–2.2)	2.5^†^ (2.0–3.0)	2.8^†^ (2.2–3.3)	2.6^†^ (2.1–3.0)	2.6 (2.2–3.0)	2.6 (2.3–3.0)	2.4 (2.1–2.7)	1.9 (1.6–2.3)	1.4 (1.0–1.9)	1.0 (0.7–1.4)
**Total, % (95% CI)**												
**2019**	**0.7** **(0.3–1.0)**	**0.7** **(0.4–1.0)**	**0.9** **(0.5–1.2)**	**1.2** **(0.9–1.5)**	**1.4** **(1.2–1.6)**	**1.6** **(1.4–1.8)**	**1.7** **(1.4–1.9)**	**1.8** **(1.6–2.0)**	**1.6** **(1.3–1.9)**	**1.2** **(1.0–1.4)**	**0.8** **(0.6–1.1)**	**0.7** **(0.4–1.0)**
**2020**	**0.8** **(0.4–1.2)**	**0.9** **(0.5–1.2)**	**1.3^†^** **(0.8–1.8)**	**2.2^†^** **(1.7–2.6)**	**2.3^†^** **(1.9–2.7)**	**2.1^†^** **(1.8–2.4)**	**2.1^†^** **(1.8–2.4)**	**2.1^†^** **(1.9–2.4)**	**1.8** **(1.6–2.1)**	**1.4** **(1.1–1.7)**	**1.0^†^** **(0.7–1.3)**	**0.8** **(0.5–1.1)**
**ED visits, no. **												
**2019**												
Pedal cyclist ED visits	13,954	12,984	19,228	25,958	33,993	38,565	42,655	44,668	38,422	26,965	17,165	14,348
Injury-related ED visits	2,067,565	1,879,084	2,196,156	2,180,453	2,386,593	2,352,969	2,543,118	2,479,241	2,425,673	2,289,238	2,046,299	2,063,942
**2020**												
Pedal cyclist ED visits	16,613	17,471	22,592	27,920	42,471	43,696	44,090	45,058	36,447	27,932	18,683	13,658
Injury-related ED visits	2,134,526	2,038,440	1,729,883	1,288,572	1,839,591	2,062,185	2,138,277	2,142,528	1,999,441	1,987,562	1,814,376	1,711,755

**Abbreviation:** ED = emergency department.

* Dashes indicate estimate suppressed because coefficient of variation >30%.

^†^ Difference in the pairwise comparison of the monthly percentage in 2020 compared with 2019 is statistically significant at p<0.05.

Although the monthly proportions of injury-related ED visits accounted for by pedal cyclist injuries were consistently higher among males in both 2019 and 2020, increases in the proportions of pedal cyclist ED visits during March–May and July–August 2020 were higher among females than males. For example, in April 2020, pedal cyclist injuries accounted for 1.7% of injury-related ED visits among females, which was 2.4 times as high as those in April 2019 (0.7%). The proportion of pedal cyclist ED visits among males in April 2020 (2.5%) was 1.6 times higher than in April 2019 (1.6%). Increases among females were sustained during March–August 2020; among males, increased monthly proportions of pedal cyclist ED visits were observed during February–June 2020.

## Preliminary Conclusions and Analysis

The proportion of injury-related ED visits accounted for by pedal cyclist injuries increased in the first year of the COVID-19 pandemic; increases were largest among children and adolescents aged <18 years, adults aged ≥50 years, and females. These findings, coupled with the recent increase in the number of pedal cycling fatalities (*3*), highlight the need for additional pedal cycling safety interventions. To reduce pedal cyclist injury risk, engineering and roadway designs that incorporate safety features for pedal cyclists (e.g., bicycle lanes) can be implemented, and states and localities can consider helmet laws for pedal cyclists of all ages to increase helmet use (*5*).

## Footnotes

* VMT is a measure of distance traveled by vehicles in a given region during a specified time. Data reported to the Federal Highway Administration by states and the District of Columbia include only motorized vehicles (e.g., cars, light trucks, sport utility vehicles, motorcycles, and heavy trucks); pedal cycle travel is not included. https://www.fhwa.dot.gov/policyinformation/tmguide/2022_TMG_Final_Report.pdf

† Pedal cyclist injuries are defined as injuries to a pedal cycle rider from a collision, loss of control, crash, or some other event. This category includes riders of bicycles, tricycles, mountain bikes, and unicycles. Injuries unrelated to riding a pedal cycle, such as repairing a bicycle or tripping over a bicycle are not included in this category. Injuries that occurred in traffic, not in traffic (such as in a driveway or other offroad location such as a bicycle trail), and where the location of injury was unspecified, were included in this analysis to capture all nonfatal pedal cyclist injuries. In 2020, 37% of pedal cyclist injuries occurred in traffic; 27% did not occur in traffic, and 37% had insufficient documentation to determine where the injury occurred. https://www.cdc.gov/injury/wisqars/nonfatal_help/index.html#nonfatal

§ 45 C.F.R. part 46.102(l)(2), 21 C.F.R. part 56; 42 U.S.C. Sect. 241(d); 5 U.S.C. Sect. 552a; 44 U.S.C. Sect. 3501 et seq.
